# The impact of *C. elegans* ceramide glucosyltransferase enzymes on the beneficial effects of *B. subtilis* lifespan

**DOI:** 10.17912/micropub.biology.000758

**Published:** 2023-04-05

**Authors:** Chelsey L Arvin, Zachary Sibila, Regina Lamendella, Jason Chan, Trisha Staab

**Affiliations:** 1 Marian University College of Osteopathic Medicine, Indianapolis, Indiana, USA; 2 Marian University College of Arts and Sciences, Indianapolis, Indiana, USA; 3 Biology, Juniata College, Huntingdon, Pennsylvania, USA

## Abstract

Ceramide glucosyltransferase (CGT) adds sugar moieties to ceramide, forming glucosylceramides that play roles in immune signaling, stress response, and host-bacterial interactions. Here, we examined whether mutations in *cgt* block the beneficial effects of *Bacillus subtilis* on *C. elegans* lifespan. We found that loss of *cgt-1* or *cgt-3* reduces lifespan compared to wildtype worms, but did not prevent the lifespan-extending phenotype of *B. subtilis* . However, *cgt-1(ok1045)* and *cgt-3(tm504)* did play a minor role in blocking stress resistance of 5-day old worms treated with *B. subtilis* . Further studying CGTs may elucidate potential roles of glucosylceramides in host-bacterial interaction.

**Figure 1. Loss of CGT does not impair the beneficial effects of B. subtilis:**
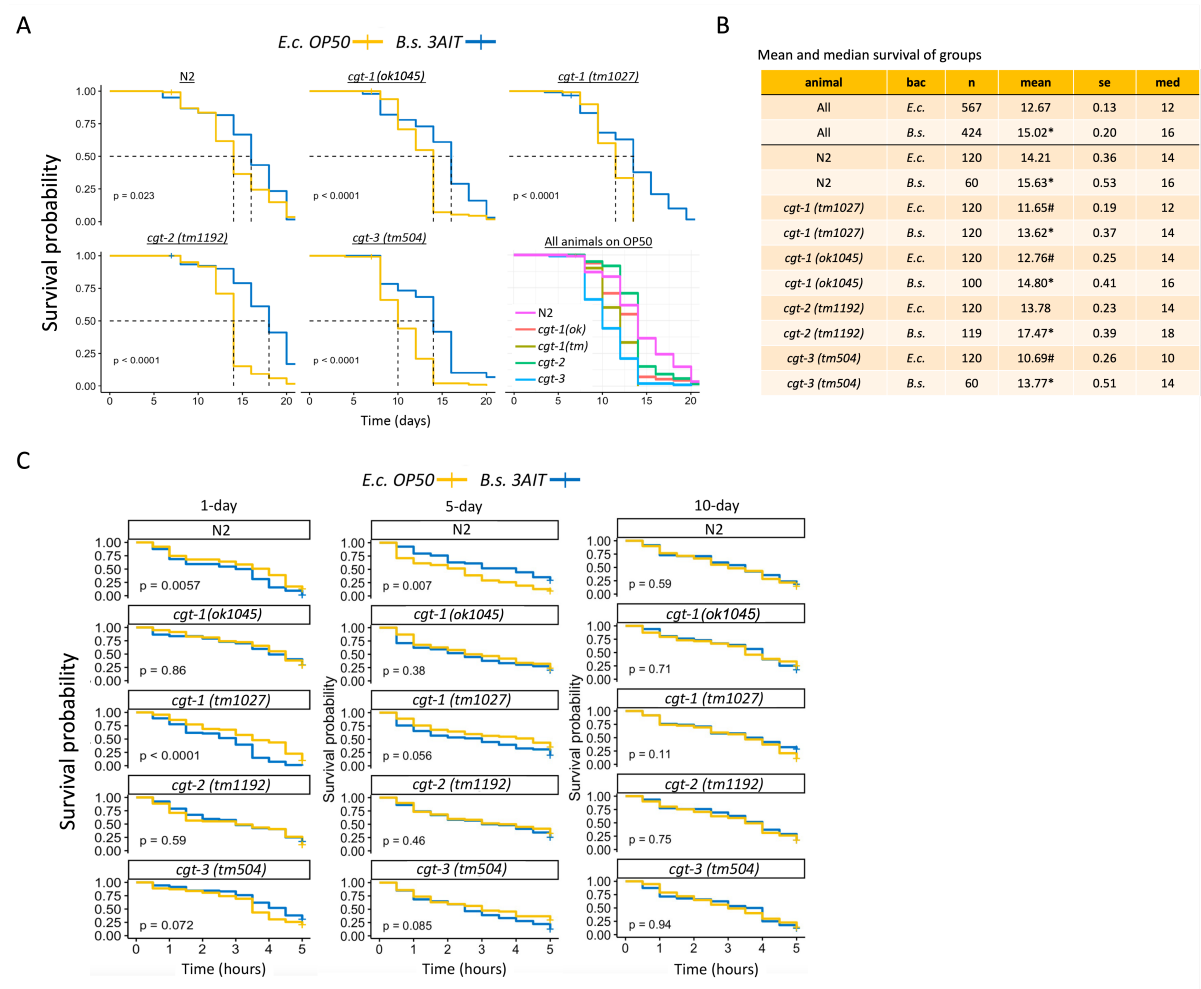
A) Survival curves of wild-type (N2) and *cgt* mutants ( *cgt-1* ( *tm1027* ), *cgt-1* ( *ok1045* ), *cgt-2* ( *tm1192* ), *cgt-3* ( *tm504* )) fed on *E. coli* (OP50; yellow) or *B. subtilis* (3A1T; blue). Worms were tracked for lifespan starting at L4 stage (day 0) and scored every 2 days. Bagged or missing worms were censored (indicated by a crossline). Significant differences were found between survival of animals treated with *E. coli* vs *B. subtilis* . The bottom right panel in (A) shows the survival curves of all animals fed *E. coli* (OP50) on the same graph. B) Table showing sample size, mean, standard error deviation, & median values for the lifespans of worms grown on the control bacteria ( *E. coli* ) and experimental bacteria ( *B. subtilis* ). For B, * indicates significant difference compared to the respective animal on *B. subtilis* and # indicates significant difference compared to N2 on *E. coli.* C) Acute stress response survival curves of wild-type (N2) and *cgt* mutants treated with 100mM paraquat. Experiments were performed on 1-day, 5-day, and 10-day old animals. Worms were grown to respective ages on either *E.coli* (OP50; yellow) or *B. subtilis* (3A1T; blue) bacterial lawns prior to the stress test. For all, survival curves were analyzed using Kaplan-Meier estimates, and pairwise comparisons were performed using a log-rank test.

## Description

At the membrane surface of intestinal cells, there is a rich complement of glucosylceramides. Ceramides are a type of sphingolipid that play a role in lipid microdomains, stress response, and cell death (Rohrhofer *et al.* 2021). The enzyme ceramide glucosyltransferase (CGT) catalyzes the addition of sugar moieties onto ceramide in the lipid bilayer. However, it is not known how bacteria-host interactions are affected by glucosylceramide metabolism. Could the presence of different *cgt* enzymes affect the impact of the beneficial effects of bacteria on the host physiology? There are three genes ( *cgt-1, cgt-2* , and *cgt-3* ) that are thought to have CGT enzymatic activity in *C. elegans* . Previous studies suggest that *cgt-1* and *cgt-3* have a greater number of amino acids relating to functional enzymatic activity; thus, mutations in *cgt-1* and *cgt-3* may have more of a negative impact on animal physiology than *cgt-2* (Marza *et al.* 2009). Specifically, *cgt-1;cgt-3* double mutants have larval phenotypes, *cgt-1* and *cgt-3* are highly expressed in the worm intestine, and they are known to serve developmental roles; furthermore, loss of all cgts ( *cgt-1, cgt-2,* and *cgt-3* ) are lethal (Marza *et al.* 2009). Re-expression of *cgt* enzymes in the intestine can rescue larval phenotypes of *cgt-1;cgt-3* double mutants, suggesting CGTs have important intestinal functions (Marza *et al.* 2009). CGTs have also been shown to help establish intestinal cell polarity during development (Zhang *et al.* 2011). More recently, there has been observation of CGTs acting on autophagolysosomes to recruit clathrin and mediate lysosome recycling (Wang et al. 2021) .

The commensal bacteria *Bacillus subtilis* has been demonstrated to increase lifespan and promote survival to the oxidative stressor juglone and thermotolerance of the nematode *Caenorhabditis elegans* (Donato *et al.* 2017; Smolentseva *et al.* 2017). Interestingly, these effects were dependent on the biofilm forming nature of *B. subtilis* . Biofilms are protective structures composed of extracellular matrix proteins and signaling molecules, providing a place for bacteria to grow and survive (Vlamakis *et al.* 2013). It also acts as a point of contact between bacteria and the host intestinal membrane. However, less is known about whether glucosylceramides mediate the beneficial effects of *B. subtilis* . Recent studies show that glycosylated ceramides are targets of *Bacillus thuringiensis* binding, leading to *C. elegans* infection (Griffitts *et al.* 2005). Furthermore, CGT inhibition can weaken the colon cell barrier to the *Bacteroides fragilis* toxin (Patterson *et al.* 2020).

Given the effect of *B. subtilis* on stress response and lifespan, along with the roles of *cgt* enzymes in the intestine, we aimed to examine whether the protective effects of *B. subtilis* require *cgt* enzymes ( *cgt-1* , *cgt-2* , and *cgt-3* ). To do this, we compared lifespan and stress response of wild-type and mutant animals when grown on either the *B. subtilis* wild-type isolate (3AIT) or the common lab bacteria *E. coli* (OP50). First, we found that *B. subtilis* increased the survival of wild-type animals approximately 10% ( Figure 1A ), which is similar to the 15% increased survival demonstrated in other studies (Donato *et al.* 2017). However, *B. subtilis* also increased the survival of all *cgt* mutant animals examined. When comparing wild-type animals to *cgt* mutants on OP50, we found that mutations in *cgt-1* and *cgt-3* reduced lifespan compared to wild-type ( Figure 1A,B ). Similarly, Wang et al. (2021) found that *cgt-3* RNAi reduced lifespan. Whereas loss of particular ceramide glucosyltransferase genes can reduce lifespan, it does not block the beneficial effects of the commensal bacteria *B. subtilis* . However, it is not clear how specific *cgt* manipulations may affect total levels of glucosylceramides. Indeed, one study showed that only double-knockout mutants ( *cgt-3;cgt-1* ) demonstrated observable phenotypes (Marza *et al.* 2009); but, another showed pharmacological inhibition of glucosylceramides, particularly glucosylceramide transferase 2, actually increase lifespan (Cutler et al. 2014) . Furthermore, the enzymes may have location- specific cell functions that have not been explored.

Previously, it was found that wild-type *B. subtilis* can promote tolerance to heavy metal, osmotic, oxidative, pathogenic, and temperature stress (Donato *et al.* 2017; Smolentseva *et al.* 2017). To examine the effect of mutations in *cgt* enzymes on adult stress response, we performed an oxidative stress assay by examining acute survival to the oxidative stressor paraquat (PQ) in 1, 5, and 10 day old animals. We found that wild-type N2 worms fed *B. subtilis* performed worse in response to 100mM PQ at 1-day old than N2 worms fed OP50 ( Figure 1C ). This was also observed in *cgt-1(tm1027)* mutants. However, 5-day-old N2 worms fed *B. subtilis* improved acute survival to PQ compared to those who fed OP50 ( *p* =0.007; Figure 1C ); however, this effect was not observed at 10-days of age. The improved response to PQ in 5-day old wild-type animals was not observed in the presence of *cgt* mutations.

In summary, we show that loss of individual CGTs impact does not block the lifespan extending effects of the bacteria *B. subtilis* . Prior research demonstrates that single CGT knockouts may play a minor role in *C. elegans* response to stress (Marza *et al.* 2009). However, others have found that *cgt-3* RNAi alone can reduce survival to the oxidative stressor TBHP (Wang et al. 2021) . Thus, further experiments deleting or knocking down multiple CGTs may provide further insight into the roles of glucosylceramides in host-bacterial interactions. It was interesting that there were some differences between 1 day and 5 day responses of wild-type animals fed OP50 vs *B. subtilis* to PQ (from a slightly detrimental effect to a slightly beneficial effect), which may suggest that colonization of *B. subtilis* is needed to impact hosts. Although speculative, this finding supports the model that biofilm formation in *B. subtilis* is necessary to promote host lifespan and physiology (Donato *et al.* 2017; Smolentseva *et al.* 2017). Nevertheless, our data suggests that CGTs may play minor roles in the beneficial effects of the commensal bacteria *B. subtilis* , unlike findings from pathogenic bacteria. Future work examining how sphingolipid enzymes affect intestinal membranes may inform our understanding of how bacteria, including those that form biofilms, impact host physiology.

## Methods

**Strains.** Wild-type N2 animals were obtained from the Caenorhabditis Genetics Center (CGC), which is funded by NIH Office of Research Infrastructure Programs (P40 OD010440). Mutants for *cgt-1(tm1027)* , *cgt-2(tm1192)* , *cgt-3(tm504)* were obtained from the Mitani lab at the National BioResource Project and *cgt-1(ok1045)* was obtained from the Caenorhabditis Genetics Center (CGC). Strains were not backcrossed into lab strains of N2. All *tm* strains are thought to eliminate CGT function or enzymatic activity (Marza *et al.* 2009). *ok1045* is a large ~1800 deletion spanning 7 exons (Wormbase). All worms were maintained on Nematode Growth Medium (Stiernagle 2006) and maintained at 20℃ on respective bacteria.

**Table:** 

Gene Name	Allele	Sequence Name	Strain	Source
*cgt-1*	tm1027	T06C12.10	JPC21	NBRC
*cgt-1*	ok1045	T06C12.10	JPC22	CGC
*cgt-2*	tm1192	F20B4.6	JPC23	NBRC
*cgt-3*	tm504	F59G1.1	JPC24	NBRC
wildtype			N2	CGC

**Bacterial cultures.** *E. coli* was obtained from the CGC and *B. subtilis* from the *Bacillus* Genetic Stock Center (BGSC). To make *E. coli* cultures, a single isolate of *E. coli* OP50 was inoculated in LB broth and incubated at 37℃. When the OD was around 0.5 (approximately 24 hours), the solution was stored at 4°C. For *B. subtilis* cultures *,* a single isolate of *B. subtilis* (3AIT) was inoculated nutrient broth and incubated at 32℃ with 125 RPM shaking. When the OD was around 0.5 (approximately 48 hours), the solution was stored at 4°C. Both OP50 and 3AIT bacteria were seeded on NGM or NGM plates supplemented with 50µM FUdR.

**Lifespan Assay** . L4 animals were placed on NGM plates supplemented with 50 µM FUdR. Lifespan measurements were made every two days and bagged/missing worms were censored on corresponding days. The plates were kept at 20℃. Animals were transferred to new 50 µM FUdR NGM plates with their respective bacteria every four days. Statistical tests for lifespan were analyzed using Kaplan-Meier survival estimates and log-rank tests, with Bonferroni correction, in the R (version 4.0) statistical package survival (version 3.5-0) and survminer (version 0.4.9).

**Acute Paraquat Assay.** N2 and *cgt* mutants at L4 stage were placed onto 50 µM FUdR NGM plates seeded with either *E. coli* (OP50) or *B. subtilis* (3AIT) and kept at 20℃. On days 1 (24 hours after L4), 5 and 10, around 60 animals of each group were transferred into 15 µL of M9 buffer in a 96 well plate (10 animals per well in six replicates). Fifteen microliters of 200 mM paraquat were then added for a final test concentration of 100 mM paraquat in each well. Animals in each well were checked for survival every 30 minutes, for five hours. Survival was determined by movement to gentle prodding. Statistical tests for survival to PQ were analyzed using Kaplan-Meier estimates and log-rank tests, with Bonferroni correction, in the R (version 4.0) statistical package survival (version 3.5-0) and survminer (version 0.4.9).

## Reagents

**Table:** 

**Product**	**Product** **Number**	**Source**
paraquat	856177	Sigma
FUdR	F0503	Sigma
